# The determinants of technical efficiency of a large scale HIV prevention project: application of the DEA double bootstrap using panel data from the Indian Avahan

**DOI:** 10.1186/s12962-015-0031-2

**Published:** 2015-03-29

**Authors:** Aurélia Lépine, Anna Vassall, Sudhashree Chandrashekar

**Affiliations:** Social and Mathematical Epidemiology (SAME) Group, Department of Global Health and Development, London School of Hygiene and Tropical Medicine, 15-17 Tavistock Place, London, WC1H 9SH UK; St. John’s Research Institute, Department of Epidemiology and Biostatistics, St. John Nagar, Bangalore, 560034 India

**Keywords:** HIV prevention, Scale-up, Two-stage DEA, Double bootstrapping, India

## Abstract

**Background:**

In 2004, the largest HIV prevention project (Avahan) conducted globally was implemented in India. Avahan was implemented by NGOs supported by state lead partners in order to provide HIV prevention services to high-risk population groups. In 2007, most of the NGOs reached full coverage.

**Methods:**

Using a panel data set of the NGOs that implemented Avahan, we investigate the level of technical efficiency as well as the drivers of technical inefficiency by using the double bootstrap procedure developed by Simar & Wilson (2007). Unlike the two-stage traditional method, this method allows valid inference in the presence of measurement error and serial correlation.

**Results:**

We find that over the 4 years, Avahan NGOs could have reduced the level of inputs by 43% given the level of outputs reached. We find that efficiency of the project has increased over time. Results indicate that main drivers of inefficiency come from the characteristics of the state lead partner, the NGOs and the catchment area.

**Conclusion:**

These organisational factors are important to explicitly consider and assess when designing and implementing HIV prevention programmes and in setting benchmarks in order to optimise the use and allocation of resources.

**JEL classifications:**

C14, I1

**Electronic supplementary material:**

The online version of this article (doi:10.1186/s12962-015-0031-2) contains supplementary material, which is available to authorized users.

## Background

The UNAIDS strategic investment framework for an effective response to HIV/AIDS proposes the scale-up of HIV prevention for key populations as one of its core interventions [1]. However, resources to expand HIV prevention to all who may benefit for it remain scarce. Due to the recent flat-lining of development assistance for health; increased attention has been placed on identifying potential efficiency gains in HIV prevention in low and middle income countries, in order to ensure value for money [2].

To date, the literature on the level and determinants of technical efficiency of HIV-related interventions is almost non–existent, but suggests room from improvement in HIV programme efficiency. Among 52 low and middle income countries [3], the technical efficiency of mother-to-child HIV prevention was 62.5% in 2008 and there was a high variability between countries (13.2% in Bolivia, 100% for 19 countries including India). Zeng et al. [4] investigate the determinants of technical efficiency in facility based HIV/AIDS services (voluntary counselling and testing, prevention of mother to child transmission and antiretroviral treatment) in a sample of low and middle income countries. They find an important disparity of efficiency level between the countries, and that even average countries could increase the outputs by 50% given their level of inputs. In China, Cheng et al. [5] measured the technical efficiency of provinces covered by the Global Fund China that provides Voluntary Counselling and Testing (VCT). They find that on average, efficiency has not increased much over time and that in the last year of the project, input level could still have been reduced by 2.17 times to reach the same quantity of outputs. To date however, there is no evidence on the determinants of technical efficiency of providers of HIV/AIDS prevention services that tend to be a mix of non-governmental organisation outreach and facility based service provision. The level of efficiency of large-scale HIV programmes and the extent therefore to which programme characteristics can influence technical efficiency remains unknown. This paper therefore aims to partially fill this gap in the setting of the Avahan project in India, one of the largest HIV prevention project conducted globally.

## Methods

### Study setting and data

Avahan is one of the largest HIV prevention project in the world. NGOs are provided grants by Avahan through state lead partners (SLPs) to build a relationship with key populations (female sex workers (FSWs) and high risk men who have sex with men/ transgenders (HR-MSM/TG) in order to provide HIV prevention services. The package of HIV prevention services provided includes outreach through peers, behavior change communication, condom distribution, clinical services for sexually transmitted infections (STIs), community mobilisation, advocacy and enabling environment activities. Peer educators provided services to about 25-50 people each, sharing prevention information, distributing supplies (condoms and lubricants) and providing referral for STI management. STI clinics followed standard protocols for STI management. Community mobilisation, advocacy and enabling environment activities varied across the sites and included the formation of self-help groups, various drop-in centre events, skills training, legal literacy workshops, police and stakeholder sensitization, crisis response teams and access to social entitlements. HIV prevention across four Indian states (Andhra Pradesh, Karnataka, Tamil Nadu, Maharashtra) was guided by a common minimum programme. These included a set of implementation standards for technical and managerial areas, project milestones, a common management framework, and a common set of indicators. Beyond this, there was flexibility to adapt services based on local context.

In the 4 study states, the Avahan initiative was implemented by 138 NGOs, supported by 6 state level partners (SLPs) and pan-Avahan capacity building partners (contracted by the BMGF, which also had a national level office at Delhi). SLPs provided technical assistance to develop programme strategies, developed communication materials, enhanced the expertise of NGO staff, provided supportive supervision and supported the purchase and distribution of commodities. At the national level, Avahan developed over-arching programme strategies and organised annual partners meetings to coordinate with Indian authorities. The national level office also developed and maintained a computerised monitoring and information system; provided financial oversight; and monitored programme evaluation. International and national technical assistance was primarily focused on enhancing the expertise to deliver STI services, improving interpersonal communication, and providing support for advocacy and community mobilisation.

Avahan achieved an exceptionally rapid pace of scale-up of HIV prevention services; going from a coverage of 22,000 persons covered in December 2003 to 280,000 persons reached per year in December 2007 (Bill and Melinda Gates Fundation, 2008). In total in the data we collected, we observe that 725,040 high-risk persons (female sex workers and their clients and men who have sex with men) were reached between 2004 and 2007, 177 million condoms were directly distributed by Avahan NGOs and 529,381 STI visits were provided. Extensive research has been conducted to evaluate the impact and cost-effectiveness of the Avahan programme. Pickles et al. [6] reported decline in FSW HIV prevalence and between 142 and 2092 FSW HIV infections averted per district, with two-fold to nine-fold more among FSW clients. Correspondingly, Vassall et al. [7] found a mean incremental cost per HIV infection averted of US$785 and a mean incremental cost per DALY averted of US$46. Future anti-retroviral treatment (ART) cost savings over the lifetime of the FSW cohort exposed to Avahan were estimated to be over US$ 77 million.

Avahan has produced one of the largest cost data set globally, further details can be found in Chandrashekar et al. [8]. Cost data were collected from 138 NGOs in 64 districts of 4 Indian states over 4 years from 2004 to 2007. Costs were collected from NGOs, SLPs, the BMGF Avahan office and pan-Avahan capacity building partners. Cost data was collected prospectively using a top down approach to allocate costs to NGOs and specific HIV prevention activities. Costs included all recurrent costs (personnel costs, project building and operating expenses, travel expenses, STI supplies, monitoring costs, information education & communication costs, training costs, condom supplies and indirect expenses) and capital costs (buildings, equipment, furniture, vehicles, initial training, insurance and deposits, and start-up costs). Methods for allocating costs above the NGO level to NGOs were derived based on programme records, expenditures reports and interviews with BMGF Avahan and SLP staff. At the NGO level, costs were disaggregated by activity and input type. Field visits and time-sheets were conducted in order to estimate the share of labour costs allocated to different NGO sub-activities (outreach, community mobilisation, etc.). Unpaid volunteer time was estimated by the amount of time spent on the project and calculated based on peer educator salary. Other donated goods, such as commodities were valued using market prices. In addition to the cost data, Avahan inputs and outputs were obtained from the surveillance data collected part of the Computerised Management Information system (CMIS) [9].

Every NGO partner was automatically included in the sample, allowing us to have an exhaustive sample of the NGOs in the Avahan programme over the period considered. The number of Avahan NGOs increased over time. In the first year of Avahan introduction there were only 58 NGO partners while in its fourth year of implementation, there were 117 Avahan NGOs, as NGOs joined and left the project. Consequently, 59% of the NGOs in the sample are observed over the entire period, with 28% of the NGOs entering in 2005, 9% in 2006 and 4% in 2007. The efficiency analysis is then conducted using an unbalanced panel. In addition, some information i.e. either at least one input or output was missing for 21 NGOs, justifying that the analysis is conducted on 377 NGOs. Ethical clearance was obtained from the Indian council of medical research (reference number HIV/51/102/2004-ECD-II).

### Data envelopment analysis

Technical efficiency is defined as performance of each site studied relative to an efficient technology that is represented by a frontier function. Data envelopment Analysis (DEA) is used in the paper for determining this frontier; and to assess the efficiency of each service unit. The choice of this non-parametric approach; in comparison to a stochastic frontier approach; is guided by the fact that the non-parametric approach imposes no restrictive hypothesis on the data generating process and requires very few assumptions about the technology [10]. We use the double bootstrap method developed by Simar & Wilson [11] in order to measure Avahan NGOs’ technical efficiency and to investigate the main predictors of technical inefficiency. The traditional two stage approach consists of estimating technical efficiency scores for each site in a first stage and then in a second stage to regress those scores, using a truncated model, on covariates. A serious drawback of this approach is that DEA efficiency estimates are found to be serially correlated [11], with a further source of endogeneity derived from the measurement error in the efficiency scores. In this paper, we therefore use the Simar & Wilson two-stage bootstrap procedure to correct for serial correlation and measurement error. In the first stage, bootstrapped DEA scores are derived from the data using an input orientation with multi-inputs and multi-outputs. In the second stage, using a bootstrapped truncated regression, these bias-corrected inefficiency scores are regressed on (1) the type of high-risk groups reached by NGOs, (2) SLP characteristics to investigate the effect of purchasing and funding on technical efficiency, (3) NGO’s organisational characteristics in order to introduce maturity and mismanagement in the analysis and (4) other environmental characteristics capturing potential scale and the presence of competition in the NGO catchment area. This approach allowing to correct for measurement error in technical efficiency scores and serial correlation in the DEA efficiency estimates has been mainly used to investigate the determinants of technical efficiency of the education sector [12-14], the transport sector [15], the agriculture sector [16,17] and the health sector [18] but has not been applied yet to the analysis of technical efficiency of HIV projects.

Using an input-orientation, the efficiency frontier is defined as the minimum level of inputs empirically observed for the NGOs given the level of outputs [19].

1 $$ \begin{array}{l}\hat{\theta}\left({x}_0,\ {y}_0\right)=\{ \min\ \theta\ |{y}_0\ \le {\displaystyle \sum_{i=1}^n}{\gamma}_i{Y}_i;\theta {x}_0\ \ge {\displaystyle \sum_{i=1}^n}{\gamma}_i{X}_i;\theta >0,\\  \qquad\qquad\qquad for\ \left({\gamma}_1,\dots,\ {\gamma}_n\right) \kern1em s.t.\kern1em {\displaystyle \sum_{i=1}^n}{\gamma}_i=1;\\ \qquad\qquad\quad\;\;{~}_{{\gamma}_i}\!\ge 0,\ i=1,\dots, n\}\end{array} $$

where $\widehat{\theta}$ is the estimated technical efficiency score, *y*_*i*_ is a vector of outputs and *x*_*i*_ a vector of inputs and *γ*_*i*_ is a I*1 vector of constants. $\widehat{\theta}\ \left({x}_0,\ {y}_0\right)$ measures the radial distance between (*x*_0_, *y*_0_) and $\left({\widehat{x}}^{\partial\ }\left({x}_0\Big|\ {y}_0\right),{y}_0\right)$ where ${\widehat{x}}^{\partial\ }\left({x}_0\Big|\ {y}_0\right)$ is the level of inputs the NGO should reach in order to be on the efficiency frontier with the same level of output *y*_0_ and input *x*_0_.

The procedure then applies Simar and Wilson’s Algorithm 2 and consists of 7 steps. Firstly, we estimate a bias-corrected estimation of the Shepard distance function^a^$\widehat{\widehat{\theta}}$ by subtracting the bootstrap bias estimate from the original distance function estimate $\widehat{\theta}$ as described in Simar & Wilson [20]. This first step corresponds to step 1 to 4 of Algorithm 2 as described in Simar and Wilson [11] and is operationalised by using Wilson’s FEAR software [21].

2 $$ \widehat{\widehat{\theta}}=\widehat{\theta}-\widehat{BIAS\left(\widehat{\theta}\right)} $$

Truncated maximum likelihood estimation is used to regress the scores on a set of explanatory variables, and then using a bootstrap procedure described in Simar & Wilson [22] we draw 2,000 samples from the truncated normal distribution of the estimated inefficiency scores. The concept behind bootstrapping is to approximate a true sampling distribution by constructing a pseudo-sample and re-solving the DEA model for each unit with the new data. Repeating this process many times builds a good approximation of the true distribution. We then calculate bias-corrected efficiency scores with the bootstrap results. By using 2,000 replications in the bootstrap, the coefficient correlation between the original and bias-corrected inefficiency scores is 0.9547 (p < 0.01).

Note that before proceeding to the second step of Algorithm 2 that consists of regressing the bias-corrected estimator on a set of covariates in order to investigate the determinants of technical inefficiency, we investigate the presence of outliers following the method proposed by PW Wilson [23]. The investigation of outliers is required since “deterministic frontier models have the drawbacks of not allowing random noise in the data generating process and, as a result, being very sensitive to extreme data points and outliers” [24].

Then, the second step (steps 5 to 7 of Algorithm 2) is performed on STATA and consists in regressing the bias-corrected technical inefficiency scores over a set of exogenous covariates using a bootstrapped truncated regression with 1,000 iterations in order to obtain unbiased coefficients and confidence intervals.

3 $$ \widehat{\theta}={\beta}_0+{\beta}_1, \dots,\ {\beta}_{n\ }{E}_{it} + {\varepsilon}_{it} $$

where *β*_0_ is a constant term and *β*_1_, …, *β*_*n*_ are coefficients of technical inefficiency determinants and *ε*_*it*_ is an error term.

### Computation of inefficiency scores

#### Selection of variables in DEA

Given the novelty of the area analysed, the selection of inputs and outputs does not come from the literature, but from consultation with the programme implementers and reviewing of previous programme literature [25]. We ran sensitivity analysis to ensure that our conclusion do not dramatically changed based on the inputs chosen. We consider a model with multiple outputs that include the three main services provided by Avahan NGOs: (1) *outreach services* are proxied by the number of high-risk at HIV persons reached by NGOs and by the number of condoms distributed, (2) *sexually transmitted infection* (*STI*) *services* are measured by the number of STI treated, and (3) the degree of *community mobilisation* is measured by the number of members of self-help groups. Inputs used to compute technical efficiency score include *labour inputs* measured by the number of management staff, the number of medical staff and the number of peer educators expressed in full time equivalency and *capital inputs* proxied by the number of offices, the number of drop in centres (for communities), the number of STI clinic vans, the number of static STI clinics, the number of outreach clinics and the number of referral clinics. Another input considered was the *managerial inputs* as measured by the Avahan capacity building costs. Finally, given that we were not able to obtain data on the quantity of *commodities* and given that NGOs face similar commodity prices, we proxy it by the expenditures on STI drugs.

These inputs and outputs are described in Table 1. Table 1 indicates that NGOs have a wide range of output levels, as the number of high-risk population reached vary from 20 to more than 10,000 per year; the number of STI visits per year is between 0 and 6,800; the number of condoms distributed varies between 0 and more than 3.2 million and the number of STI treated between 0 and about 10,000. Finally the number of members for community mobilization varies between 0 and 470. This indicates that at some point (especially in the first year of the intervention) some NGOs do not necessarily provide other services than outreach services. The output variables are positively correlated given that high-risk populations need to be reached to receive other outputs (such as STI visits, STI treatment and condoms). However, from Additional file 1, we can see that these variables are not highly correlated as recommended in the literature regarding the selection of variables in DEA [26,27], which also suggests that the selection of inputs and outputs does not include double counting. We therefore did not need to remove the outreach variable in the DEA. Moreover, the fact that the number of persons reached is a key activity of Avahan NGOs, justifies the inclusion of outreach services in the output list. Finally, the number of inputs and outputs (n=10) is low compared to the number of DMUs (n=387), well below the recommendations of having one-third of the number of DMUs as the number of inputs/outputs [28,29].

**Table 1:** **Key statistics for inputs and outputs (period 2004-2007)**

**Variables**	**n**	**Mean**	**Std. Dev.**	**Min**	**Max**
**Inputs**					
*Labour inputs*					
Number of medical staff	377	5.48	3.96	0	25
Number of outreach staff	377	52.58	34.09	0	220
*Equipment*					
Number of office	377	1.14	1.85	0	18
Number of Drop in Centre	377	3.27	3.66	0	17
Number of clinic van	377	0.02	0.19	0	3
Number of static clinic	377	1.69	1.87	0	15
Number of outreach clinics	377	4.25	10.14	0	69
Number of referral clinics	377	1.57	4.01	0	23
*Managerial cost*					
Capacity building cost (USD)	377	56,779	43,538	1296	331,846
*Commodities*					
Cost of STI services (USD)	377	21,784	19,243	56	150,842
**Outputs**					
Number of high risk population reached	377	1,785	1,491	20	10,119
Number of STI visits	377	1,273	1,212	0	6,804
Number of condom distributed	377	413,134	483,270	0	3,257,058
Number of STI treated	377	783	1,455	0	9,953
Number of members of self-help groups	377	23	52	0	470

#### Outlier analysis

As shown in Additional file 2, the corrected log-ratio plot has 4 peaks that correspond to *cpi*=1, 3, 6, 10 and identify four groups of outliers and a total of 10 outliers representing 8 NGOs. Supplementary file 2 indicates that observations 73 as well as 137 and 84 are outliers. Another group of outliers are observations 283, 39 and 170. A last group of outliers are observations 345, 378, 377 and 315. In order to investigate the weight of outliers units, we removed the outlier units and compare correlation coefficients, we found that the correlation coefficient was 0.995 (p < 0.01), suggesting that the removal of the outliers units will not substantial affect our results.

## Results and discussion

### Descriptive statistics of TE scores

Variable returns to scale were considered since all the NGOs may not be operating at an optimal scale. The inefficiency scores are then left-truncated and are found to vary between 1 (for efficient units) and 3.64 in the data, i.e. that the least efficient unit in the year observed could have decreased its level of inputs by 3.64 times given the level of outputs. Among the 138 NGOs in the data, 36 appear to be efficient at least one year (16 are efficient in 2004, 9 in 2005 and 11 in 2007), resulting in an average Shepard efficiency score over the 4 year period of 1.51. However, this first inefficiency score is biased as stated previously. In order to correct for measurement error, the bootstrap procedure of Simar & Wilson [11,20] is used to correct for bias in the estimates of the efficiency scores, allowing estimating at the same time the confidence intervals for the Shepard distance input function.

When we correct for bias, the overall efficiency score over the 4 year period is 1.75^b^ (instead of 1.51). Using the Farell technical efficiency, it means that the system could reduce the consumption of all inputs by 43% without reducing the level of outputs. From Table 2 we can see that the average inefficiency of the Avahan project has first increased over the first year of implementation of the programme and then decreased over 2005-2007. In the first year of Avahan, Avahan NGOs could have decreased the level of inputs by 1.88 times to reach the same level of outputs and in the last year by 1.49 times showing an increase in efficiency in Avahan. If we look at the change of efficiency over time looking at the NGOs observed over the full period (i.e. for which we have a balanced panel), we also find that average inefficiency has decreased: the corrected-shepard efficiency score was 1.98 in 2004 and 1.43 in 2007.

**Table 2:** **Corrected-Shepard efficiency scores over the first four years of Avahan**

	**n**	**Mean**	**Std. Dev.**	**Min**	**Max**
2004	58	1.883	0.627	1.170	3.420
2005	96	1.990	0.766	1.166	4.153
2006	106	1.747	0.534	1.161	3.910
2007	117	1.494	0.475	1.114	3.587

### Determinants of technical inefficiency

Several potential environmental determinants of technical inefficiency are explored, that represent key theoretical drivers of average cost [25]. These include (1) the type of high-risk population reached (female sex worker versus men who have sex with men), (2) potential scale measured here by the estimated number of high-risk populations in the catchment area,^c^ (3) competition level measured by the number of Avahan NGOs per district and by whether or not the NGO operate alone with HIV high-risk population over a district, (4) purchasing and funding characteristics proxied by the number of NGO per SLP and SLP dummies, (5) mismanagement that is measured by whether or not the NGO was replaced during the year and (6) maturity as measured by the number of years in Avahan, by whether or not the NGO has HIV experience before joining Avahan and by time dummies. The expected relationship between those environmental variables and technical inefficiency is presented in Table 3 and descriptive statistics of environmental variables are presented in Table 4.

**Table 3:** **Expected effect of environmental variables on technical inefficiency**

**Category**	**Variable name**	**Description**	**Expected effect**	**Justification**
Type of client	FSW/Y_it_	Share of female sex workers (FSW) in total reached population that includes sex workers and men who have sex with men (MSM)	-	It may be easier to reach female sex workers than MSM given the high stigma against MSM in India, then the proportion of FSW in high-risk population may decrease inefficiency
Potential scale	Log of estimated population	Estimated number of high-risk population in the district	+ or-	Acts as a barrier to NGO expansion since NGOs cannot expand beyond district. However, NGOs located in areas with many high-risk populations could also experience some logistic and management issues
Competition	NGOs per district	Number of NGOs in the district	+ or-	There could be some positive or negative externalities on the average cost depending on the density of the NGOs per district
	Solo district	Takes the value of 1 if the NGO is alone to operate in the district and the value of 0 if there is a co-intervention with other AIDS control society intervention or a non-Avahan intervention	+	Competition between NGOs of different projects may reduce inefficiency then NGO operation alone may be positively correlated with inefficiency. NGOs operating alone in a district may also be as a result of the remoteness and poor characteristics of the districts which should also be positively with inefficiency
Purchasing & funding	NGOs per SLP	Number of NGOs supported by the same SLP	+ or-	One could argue that SLP that are only contracting with a unique NGO could lack of experience, however if the number of NGOs per SLP is too high, it may be a source of managerial issue.
	SLP	SLP dummy	+ or-	Depending on the SLP characteristics
Mismanagement	NGO replaced	NGO was replaced by another NGO	+	May be a signal for high level of inefficiency. May capture corruption level and a lack of organisation.
Maturity	Years in Avahan	Number of years in the Avahan initiative	-	Gain of experience may decrease inefficiency
	HIV experience	Had HIV experience before entering Avahan	-	NGOs with experience in HIV may have already worked with high-risk groups which could decrease inefficiency
	Time	Year dummy	-	Inefficiency is likely to decrease over time

**Table 4:** **Descriptive statistics of environmental variables**

**Variables**	**n**	**Mean**	**Std. Dev.**	**Min**	**Max**
Inefficiency score (corrected)	377	1.751	0.629	1.114	4.153
Share FSW in total persons reached (%)	377	77.711	34.334	0	100
Estimated population	377	1,686.716	1,154.953	51	12,929
Estimated population squared	377	4,175,390	9,886,143	2,601	1.67E + 08
Number of NGOs per district	377	2.862	2.312	1	10
Number of NGOs per SLP	377	19.694	6.569	9	35
Number of NGOs per SLP squared	377	430.931	291.1961	81	1,225
NGO was replaced (%)	377	0.031	0.175	0	1
Duration in Avahan (in year)	377	3.458	0.811	1	4
HIV experience prior Avahan (%)	377	0.371	0.483	0	1
Solo district (%)	377	0.442	0.497	0	1
SLP 2 (ref: SLP 1) (%)	377	0.119	0.324	0	1
SLP 3 (%)	377	0.079	0.270	0	1
SLP 4 (%)	377	0.151	0.358	0	1
SLP 5 (%)	377	0.257	0.437	0	1
SLP 6 (%)	377	0.225	0.418	0	1
2005 (ref: 2004) (%)	377	0.254	0.436	0	1
2006 (%)	377	0.281	0.450	0	1
2007 (%)	377	0.310	0.463	0	1

Table 5 present the characteristics of the top 10% most and least efficient units. We find that NGOs that reach a larger share of FSW and NGOs and NGOs operating in an area where the estimated population is lower are more inefficient. We also find that the SLP and year have a substantial impact on inefficiency. In the group of most inefficient NGOs, a large proportion were observed in 2007, suggesting that the NGOs that entered the project later on were more inefficient on average. The other factors do not seem to play a role in the bivariate analysis.

**Table 5:** **Characteristics of the most and least inefficient units**

	**n=37 (least inefficient)**		**n=39 (most inefficient)**		
**Variable**	**Mean**	**Std. Dev.**	**Mean**	**Std. Dev.**	**Mean test p-value**
Inefficiency score (corrected)	3.126	0.421	1.181	0.020	<0.001
Share FSW in total persons reached (%)	71.258	37.693	85.379	28.682	0.071
Estimated population	1864.154	732.220	1411.270	703.625	0.007
Estimated population squared	3997469	3699676	2473391	2169280	0.032
Number of NGOs per district	2.897	2.245	2.621	1.976	0.572
Number of NGOs per SLP	19.153	5.392	20.486	7.928	0.392
Number of NGOs per SLP squared	395.205	191.965	480.864	385.499	0.220
NGO was replaced (%)	0.051	0.223	0.0277	0.164	0.593
Duration in Avahan (in year)	3.538	0.600	3.243	1.090	0.145
HIV experience prior Avahan (%)	0.282	0.455	0.270	0.450	0.910
Solo district (%)	0.512	0.506	0.405	0.497	0.354
SLP:					<0.001
SLP 2 (%)	0.179	0.388	0.027	0.164	
SLP 3 (%)	0.461	0.505	0.054	0.229	
SLP 4 (%)	0.179	0.388	0.351	0.483	
SLP 5 (%)			0.351	0.483	
SLP 6 (%)			0.0540	0.229	
Year:					<0.001
2005 (%)	0.512	0.506	0.135	0.346	
2006 (%)	0.179	0.388	0.162	0.373	
2007 (%)	0.153	0.365	0.540	0.505	

Table 6 suggests that the most important determinants of technical inefficiency are the state level partner that has contracted with the NGO; time; whether or not the NGO is operating alone in the district; whether or not the NGO was replaced; whether or not the NGO had previous experience in the provision of HIV service; the number of NGOs per SLP and the size of the estimated population.

**Table 6:** **Determinants of technical efficiency (truncated regression)**

	**β**	**SE**	**95% bootstrap confidence intervals**	
			**Low**	**High**
Share FSW in total persons reached	−0.006***	0.002	−0.010	−0.003
Estimated population	0.001***	0.0002	0.0001	0.001
Estimated population squared	−7.66E-08**	4.31E-08	−1.72E-07	−3.08E-09
Number of NGOs per district	−0.090**	0.054	−0.201	0.010
Number of NGOs per SLP	0.186**	0.102	−0.013	0.385
Number of NGOs per SLP squared	−0.003*	0.002	−0.007	0.001
NGO was replaced	0.500*	0.323	−0.239	1.027
Duration in Avahan	0.123	0.116	−0.096	0.357
HIV experience prior Avahan	0.301**	0.144	−0.002	0.563
Solo district	0.329**	0.162	0.017	0.652
SLP 2 (ref: SLP 1)	2.013***	0.472	1.043	2.893
SLP 3	2.623***	0.530	1.537	3.616
SLP 4	0.662**	0.347	−0.052	1.307
SLP 5	1.204***	0.340	0.466	1.801
SLP 6	0.842***	0.294	0.218	1.372
2005 (ref: 2004)	−0.115	0.216	−0.529	0.316
2006	−0.782***	0.262	−1.221	−0.195
2007	−1.550***	0.309	−2.064	−0.851
Observations	377			
Number of NGOs	130			

***p < 0.01, **p < 0.05, *p < 0.1 Confidence intervals obtained from 1,000 bootstrapping iterations.

To test multicollinearity, Variance Inflation Factors (VIF) are used. The VIF shows how much the variance of the coefficient estimate is being inflated by multicollinearity. The largest VIF was 2.46 and the mean VIF was around 1.52, which does not suggest high multicollinearity.

Multivariate analysis confirms that inefficiency has decreased over time probably due to the learning by doing of NGOs. This result is interesting given that in a previous paper analysing the determinants of average cost [25], we found that average cost has increased over time. However, in that paper we were unable to disentangle the effect of input price increases and technical efficiency. This finding suggests that technical efficiency has increased over time, and thus suggests that any increase in cost may be due to an above inflation increase in input prices. This is plausible, particularly as Avahan was scaled up so rapidly, that the demand on HIV specific resources, such as skilled staff may have been inflationary.

We find that NGOs that operate alone in their district are more inefficient, maybe either because a lack of competition may increase their inefficiency or because efficient NGOs are less willing to work in a remote and poor catchment area. Correspondingly, we also find that when the number of NGOs per district increases, it reduces inefficiency, then reinforcing the hypothesis that competition may increase the efficiency of Avahan. Here competition may play a role since it would increase the choice for the SLP in case a particular NGO does not do well, SLP can request another NGO working in the same district (taluka) to widen their coverage and discontinue working with inefficient NGOs. We also find that NGOs that have been replaced are more likely to be inefficient as expected.

The SLP is a key factor determining technical efficiency, corresponding with our previous finding that the SLP is a key driver of cost [25]; potentially illustrating the important role of support and supervision in achieving efficiency. Additionally, the number of NGOs per SLP is positively correlated with inefficiency when the number of NGOs per SLP is below 30. However, it then increases efficiency when there are more than 30 NGOs per SLP (which is only the case if the NGOs that contracted with the SLP6 in 2007). This suggests that a higher number of NGOs per SLP may result in organisational challenges and the finding may indicate that SLPs do not have the ability to contract with many NGOs.

We also find that NGOs with previous HIV experience do not perform better as expected. In fact, we find a positive relationship between this variable and inefficiency suggesting that NGOs with prior experience in HIV are likely to be less efficient than NGOs with no experience. It is conceivable that NGOs lacking experience will be more open to learning and experimenting than the experienced ones that may be more resistant to the innovative or fresh ideas of Avahan programming. We find that although efficiency of Avahan has increased over time, we do not find that younger Avahan NGOs are doing worse. This may be explained by the fact that NGOs benefit from externalities from other NGOs when they enter in Avahan after its first year of implementation. Additionally, various capacity building activities have been implemented to handhold the inexperienced NGOs.

A further finding is that we find that a greater number of high-risk persons in the catchment area increases inefficiency, given this finding opposes our previous finding of the presence of economies of scale highlighted in a previous paper that examines the relationship between the numbers of persons reached by HIV services and costs [25].

We also find that the type of high-risk population targeted has little effect on NGO’s technical efficiency as an increase in one percent of the proportion of FSW in total persons reached reduces the inefficiency score by only 0.006 points.

## Discussion

We used the double bootstrapping method of Simar and Wilson to investigate the determinants of technical efficiency of Avahan in India, one of the largest HIV prevention project conducted so far. We find that over the 4 years of scale up of the Avahan initiative, the overall level of inefficiency suggests that Avahan NGOs could have reduced their level of inputs by 1.75 times (or 43%) given the level of outputs reached. However, inefficiency was reduced substantially over time, since in the last year of Avahan scale-up (in 2007), NGOs could have reduced the level of inputs by 1.49 times. It is hard to place these levels in a broader context, due to the dearth of information on technical efficiency of HIV projects. However, if we compare Avahan to the only other HIV prevention intervention with information on technical efficiency [5], we find that Avahan is much more efficient.

The method we use corrects for measurement error in technical efficiency scores and for the fact that those scores are serially correlated. Although the selection of inputs and outputs has been done with programme implementers, the main limitation of the paper is that we were not able to have a better measure of quality for the different outputs. In the context of Avahan, quality was proxied by the diversity of services offered to high-risk group such as the treatment of STI and the community mobilisation. However we do not have any exogenous measure of quality of the outreach service and STI treatment. Another limitation comes from the absence of information on the population reached. In fact, one may want to argue that factors such as the level of education and income of the population reached may explain variability in efficiency.

The results have important policy implications. Firstly, regarding the selection of NGOs we find that NGOs with previous general work experience on HIV are no more efficient than those without experience. This suggests that for these services policy makers should be open to attracting NGOs from different sectors. Another important element regarding the selection of NGOs is that the replacement of an inefficient NGO has a long term negative effect on technical efficiency. In fact, NGOs that have replaced inefficient NGOs are also found to be less efficient on average. It usually takes time for the new NGO to build rapport with the high-risk groups and the staff turnover issues and start up time for the NGO could increase inputs or reduce outputs in that year. This suggests that careful attention should be paid when selecting NGOs to scrutinise and pre-assess general management capacity and financial solvency.

Regarding project implementation, our findings suggest that competition between NGOs may be an important determinant of technical efficiency, as having several NGOs operating in the same area can help improving the overall efficiency of large scale HIV prevention projects. This is an interesting finding given that previous studies have shown the importance of economies of scale when scaling up HIV services [30]. High economies of scale have been confirmed in the context of Avahan [25], justifying that efficiency could be improved by increasing the size of NGOs rather than increasing their number. The importance of competition in Avahan efficiency shows that there needs to be a careful balance between achieving economies of scale while not stifling competition.

Secondly, our findings suggest that the way in which NGOs assess and measure their population in need matters for the programme efficiency. This highlights the importance in supporting sound planning processes; and this policy recommendation is further supported by the fact the SLP has an important effect on NGO technical efficiency. As SLPs had a grant based method of contracting with NGOs and there was no formal incentive provided to support efficiency, the effect of SLP on technical efficiency may then be attributable to informal incentives and to the quality of support SLPs give to NGOs.

Finally, given that technical efficiency has increased over time, it could be that efficiency gains are generated thanks to learning by doing. This assumption is supported by the fact that NGOs entering the project in later years do seem to benefit from positive externalities from experienced Avahan NGOs, possibly due to scale of programme influence and efforts to disseminate programme learnings broadly; and suggests that many of the drivers in inefficiency lie within the control of programme managers and implementers.

## Conclusion

Using the double bootstrap method we measured the technical efficiency of Avahan, one of the largest HIV prevention project in the world and identified a number of strategies regarding the selection of NGOs and implementation of Avahan that could allow improving the technical efficiency of the project. These organisational factors are important to explicitly consider and assess when designing and implementing HIV prevention programmes and in setting benchmarks in order to optimise the use and allocation of resources.

## Endnote

^a^Note that the Shephard’s input distance function is the reciprocal of Farrell’s measure of technical efficiency and thus a proxy for inefficiency, a greater score being associated with greater inefficiency.

^b^100 − (1/1.75 * 100).

^c^Note that this has been estimated by epidemiologists.

## Additional files

Additional file 1:
**Correlation of variables included in the DEA (n=377).**
Additional file 2:
**Treatment of outliers.**
