# Supplementary material for: The determinants of technical efficiency of a large scale HIV prevention project: application of the DEA double bootstrap using panel data from the Indian Avahan
Source: Cost Eff Resour Alloc. 2015 Mar 29;13:5. doi: 10.1186/s12962-015-0031-2 (PMC4389319; doi:10.1186/s12962-015-0031-2)
Supplement: Additional file 1: — Correlation of variables included in the DEA (n=377). [file 12962_2015_31_MOESM1_ESM.docx]

Supplementary file 1: correlation of variables included in the DEA (n=377)

**Inputs**

|  | Number of medical staff | Number of outreach staff | Number of office | Number of Drop in Centre | Number of clinic van | Number of static clinic | Number of outreach clinics | Number of referral clinics | Capacity building cost | Cost of STI services |
| --- | --- | --- | --- | --- | --- | --- | --- | --- | --- | --- |
| *Labour* |  |  |  |  |  |  |  |  |  |  |
| Number of medical staff | 1.00 |  |  |  |  |  |  |  |  |  |
| Number of outreach staff | 0.46 | 1.00 |  |  |  |  |  |  |  |  |
| *Equipment* |  |  |  |  |  |  |  |  |  |  |
| Number of office | 0.35 | 0.14 | 1.00 |  |  |  |  |  |  |  |
| Number of Drop in Centre | 0.38 | 0.35 | 0.33 | 1.00 |  |  |  |  |  |  |
| Number of clinic van | 0.15 | 0.04 | -0.01 | 0.02 | 1.00 |  |  |  |  |  |
| Number of static clinic | 0.34 | 0.50 | 0.65 | 0.09 | 0.09 | 1.00 |  |  |  |  |
| Number of outreach clinics | 0.18 | 0.11 | 0.00 | 0.22 | -0.04 | 0.01 | 1.00 |  |  |  |
| Number of referral clinics | -0.08 | 0.08 | 0.15 | 0.51 | -0.04 | 0.27 | -0.05 | 1.00 |  |  |
| *Managerial input* |  |  |  |  |  |  |  |  |  |  |
| Capacity building cost | 0.16 | 0.23 | 0.06 | 0.25 | 0.02 | 0.21 | 0.24 | 0.03 | 1.00 |  |
| *Commodities* |  |  |  |  |  |  |  |  |  |  |
| Cost of STI services | 0.35 | 0.61 | 0.18 | 0.42 | -0.03 | 0.37 | 0.27 | 0.25 | 0.43 | 1.00 |

**Outputs**

|  | Number of high risk population reached | Number of STI visits | Number of condom distributed | Number of STI treated | Number of members of self-help groups | | |
| --- | --- | --- | --- | --- | --- | --- | --- |
| Number of high risk population reached | 1 |  |  |  |  |  |  |
| Number of STI visits | 0.7601 | 1 |  |  |  |  |  |
| Number of condom distributed | 0.5444 | 0.5762 | 1 |  |  |  |  |
| Number of STI treated | 0.4389 | 0.5934 | 0.2369 | 1 |  |  |  |
| Number of members of self-help groups | 0.3406 | 0.337 | 0.5072 | 0.1554 |  | 1 |  |
