# Supplementary material for: The determinants of technical efficiency of a large scale HIV prevention project: application of the DEA double bootstrap using panel data from the Indian Avahan
Source: Cost Eff Resour Alloc. 2015 Mar 29;13:5. doi: 10.1186/s12962-015-0031-2 (PMC4389319; doi:10.1186/s12962-015-0031-2)

Supplementary file 2: Treatment of outliers

Outlier observations

| *i* | *Observations* | | | | | | | | | | | | | *R^(i)^_min_* |
| --- | --- | --- | --- | --- | --- | --- | --- | --- | --- | --- | --- | --- | --- | --- |
| [1] | 73 |  |  |  |  |  |  |  |  |  |  |  |  | 0.42451 |
| [2] | 73 | 137 |  |  |  |  |  |  |  |  |  |  |  | 0.208072 |
| [3] | 73 | 137 | 84 |  |  |  |  |  |  |  |  |  |  | 0.088067 |
| [4] | 73 | 137 | 84 | 283 |  |  |  |  |  |  |  |  |  | 0.050995 |
| [5] | 73 | 137 | 84 | 283 | 39 |  |  |  |  |  |  |  |  | 0.027837 |
| [6] | 73 | 137 | 84 | 283 | 39 | 170 |  |  |  |  |  |  |  | 0.012781 |
| [7] | 73 | 137 | 84 | 283 | 39 | 170 | 345 |  |  |  |  |  |  | 0.007379 |
| [8] | 73 | 137 | 84 | 283 | 39 | 170 | 345 | 378 |  |  |  |  |  | 0.004456 |
| [9] | 73 | 137 | 84 | 283 | 39 | 170 | 345 | 378 | 377 |  |  |  |  | 0.00263 |
| [10] | 73 | 137 | 84 | 283 | 39 | 170 | 345 | 378 | 377 | 315 |  |  |  | 0.001651 |
| [11] | 73 | 137 | 84 | 283 | 39 | 170 | 345 | 378 | 377 | 315 | 130 |  |  | 0.001134 |
| [12] | 73 | 137 | 84 | 283 | 39 | 170 | 345 | 378 | 377 | 315 | 130 | 334 |  | 0.000809 |

Log-ratio plot


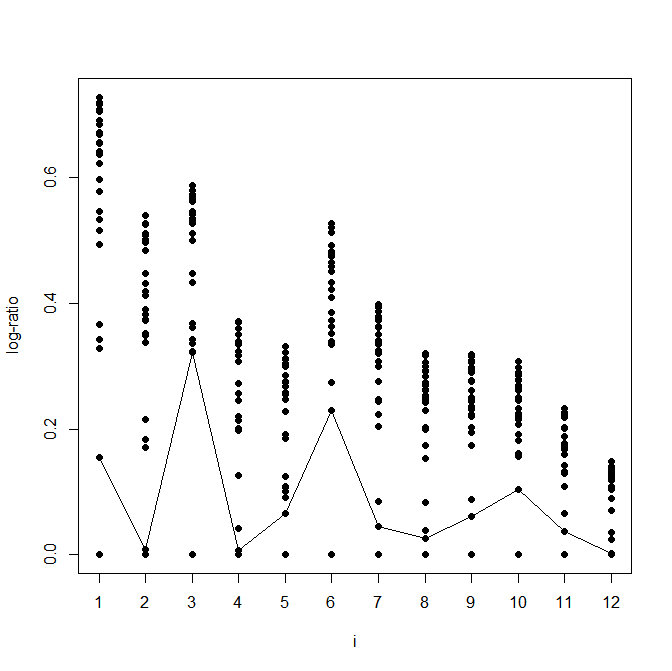

Supplement: Additional file 2: — Treatment of outliers. [file 12962_2015_31_MOESM2_ESM.docx]
